# Echinacoside's nigrostriatal dopaminergic protection against 6‐OHDA‐Induced endoplasmic reticulum stress through reducing the accumulation of Seipin

**DOI:** 10.1111/jcmm.13285

**Published:** 2017-08-02

**Authors:** Yajie Zhang, Hongyan Long, Fuqiong Zhou, Weina Zhu, Jie Ruan, Yang Zhao, Yan Lu

**Affiliations:** ^1^ Central Laboratory The Third Affiliated Hospital of Nanjing University of Chinese Medicine Nanjing Jiangsu China; ^2^ Clinical Biobank of Nanjing Hospital of Chinese Medicine The Third Affiliated Hospital of Nanjing University of Chinese Medicine Nanjing Jiangsu China; ^3^ Department of Pediatrics The Third Affiliated Hospital of Nanjing University of Chinese Medicine Nanjing Jiangsu China; ^4^ Institute of T.C.M. The Third Affiliated Hospital of Nanjing University of Chinese Medicine Nanjing Jiangsu China; ^5^ Department of Neurology The Third Affiliated Hospital of Nanjing University of Chinese Medicine Nanjing Jiangsu China

**Keywords:** Parkinson's disease, 6‐OHDA, Echinacoside, Seipin, Nigrostriatum, endoplasmic reticulum stress

## Abstract

Parkinson's disease (PD) is one of the most common neurodegenerative diseases. Recent epidemiological studies suggest that echinacoside (ECH), a phenylethanoid glycoside found in *Cistanche deserticola*, has a protective effect against the development of PD. However, the detailed mechanisms of how ECH suppresses neuronal death have not been fully elucidated. In this study, we confirmed that ECH protects nigrostriatal neurons against 6‐hydroxydopamine (6‐OHDA)‐induced endoplasmic reticulum stress (ERS) *in vivo* and *in vitro*. ECH rescued cell viability in damaged cells and decreased 6‐OHDA‐induced reactive oxygen species accumulation *in vitro*. It also rescued tyrosine hydroxylase and dopamine transporter expression in the striatum, and decreased α‐synuclein aggregation following 6‐OHDA treatment *in vivo*. The validated mechanism of ECH activity was the reduction in the 6‐OHDA‐induced accumulation of seipin (Berardinelli–Seip congenital lipodystrophy 2). Seipin has been shown to be a key molecule related to motor neuron disease and was tightly associated with ERS in a series of *in vivo* studies. ECH attenuated seipinopathy by promoting seipin degradation *via* ubiquitination. ERS was relieved by ECH through the Grp94/Bip‐ATF4‐CHOP signal pathway.

## Introduction

PD, the second most common neurodegenerative disorder after Alzheimer's disease (AD), is characterized by slow progressive degeneration of dopamine (DA) neurons in the substantia nigra pars compacta (SNpc) 1, 2. When symptoms of PD (such as resting tremor, bradykinesia, rigidity and postural instability) appear, at least 50% of all nigral neurons have degenerated and striatal DA levels have reduced by 80% 3. Although the neuropathological hallmarks of PD are well described, the aetiology remains still undefined. Mitochondrial dysfunction and oxidative stress 4, 5, 6, abnormal protein accumulation and aggregation 7, 8, impaired protein degradation *e.g*. the autophagy‐lysosomal pathway 9, 10 and ubiquitin‐proteasome pathway 11 and inflammatory responses 12 are all involved in the mechanism of cell damage in PD.

Endoplasmic reticulum (ER) dysfunction has an important part to play in a range of neurological disorders, including PD 13. Thus, drugs that interfere with ER stress (ERS) have great therapeutic potential. Scientists have investigated the effects of drugs on the following three arms of ER stress: the protein kinase RNA‐activated (PKR)‐like ER kinase (PERK) arm, the activated transcription factor 6 (ATF6) arm and the inositol‐requiring enzyme 1 (IRE1) arm 14. Increasing evidence from human and animal studies has suggested that seipin, an ER‐resident membrane protein, plays an important role in motor neuron diseases 15, and activates the unfolded protein response (UPR) pathway and induces ERS‐mediated cell death 16.

**Figure 1:**
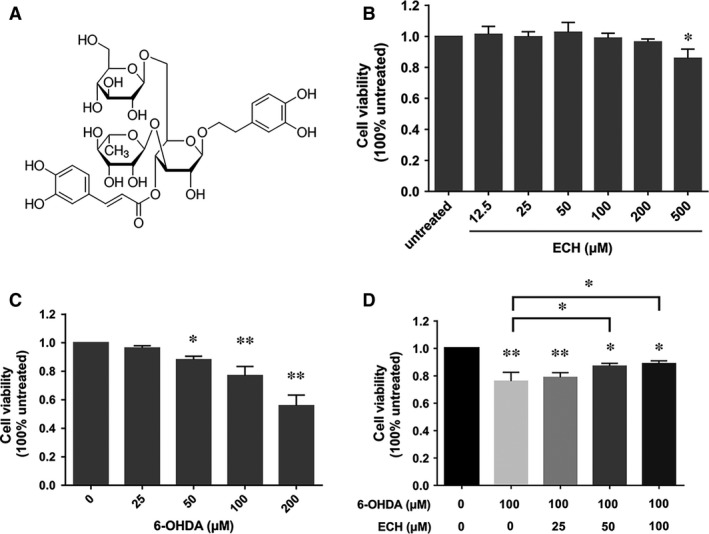
Protective effect of ECH on viability of PC12 cells injured by 6‐OHDA. Cell viability was determined by MTT assay. Data are expressed as percentage of the viability of cells, control taken as 100% viability. (**A**) The chemical structure of echinacoside (ECH). (**B**) Effect of different concentration of ECH on cell viability in PC12 cells (determine the non‐toxic dosages of ECH). PC12 cells were incubated with ECH (12.5–500 μM) for 24 hrs. (**C**) Effect of different concentration of 6‐OHDA on cell viability in PC12 cells (determine the toxic dosages of 6‐OHDA). PC12 cells were treated with various concentrations of 6‐OHDA (0–200 μM) for 24 hrs. (**D**) Effect of ECH on cell viability changes in 6‐OHDA‐induced PC12 cells. PC12 cells were pre‐incubated with different concentration of ECH (0, 25, 50, 100 μM) for 1 hr. Then, 6‐OHDA was added to the wells at a final concentration of 100 μM and incubated for another 24 hrs at 37°C. Data were shown as mean ± S.D.,*n* = 4, *P < 0.05, **P < 0.01

ECH (Fig. 1A) is a phenylethanoid glycoside isolated from the stems of the traditional Chinese medicine, *Cistanche salsa*, which possesses a spectrum of beneficial activities including neuroprotective, hepatoprotective, anti‐inflammatory, anti‐fatigue, antineoplastic and antioxidant effects 17. Previous studies have reported that ECH could protect striatal dopaminergic neurons against injury induced by 6‐OHDA through attenuating mitochondrial dysfunction and inflammatory responses by reducing ROS production 18. However, it is still not clear whether the neuroprotection of ECH is associated with its effect on ERS or seipin.

6‐OHDA is a selective dopaminergic neurotoxin 19, which is specific to dopaminergic neurons in rodent models, that produces reactive oxygen species (ROS) 20. Injection of 6‐OHDA into the striatum may produce a continuous lesion after several minutes to 1 week or month that will finally lead to the emergence of PD‐like symptoms. The PC12 cell line is widely used to investigate cellular functions in neurodegenerative diseases 21. Therefore, we used PC12 cells and 6‐OHDA‐lesioned rats as *in vitro* and *in vivo* models of PD, respectively.

In this study, we attempted to investigate the mechanism of the protective effect of ECH against 6‐OHDA‐induced ERS in nigrostriatal dopaminergic neurons *via* reducing accumulation of seipin and aimed to observe the effects of ECH on the UPR pathway.

## Materials and methods

### Materials

ECH (CAS No. 82854‐37‐3, PubChem CID:5281771), 6‐OHDA, DA, 3,4‐dihydroxyphenylacetic acid (DOPAC), homovanillic acid (HVA), apomorphine, 3‐(4,5‐dimethylthiazol‐z‐yl)‐2,5‐diphenyltetrazolium (MTT) and 2′,7′‐dichlorofluorescin diacetate (DCFH‐DA) were purchased from Sigma‐Aldrich (St. Louis, MO, USA). RPMI‐1640 and foetal bovine serum were obtained from Gibco (Grand Island, NY, USA). The primary antibodies used in the study were as follows. Anti‐seipin (Berardinelli–Seip congenital lipodystrophy 2, BSCL2) rabbit antibody was purchased from Abcam (Cambridge, MA, USA. Catalog: ab106793). Anti‐seipin goat antibody was purchased from Santa Cruz (Dallas, TX, USA. Catalog: sc‐55987). Anti‐tyrosine hydroxylase (TH) antibody (ab75875), anti‐GRP94 (ab108606) antibody, anti‐GRP78/Bip antibody (ab108613) and anti‐ATF4 antibody (ab50546) were purchased from Abcam. Anti‐dopamine transporter (DAT) antibody was purchased from Proteintech (Wuhan, Hubei, China. Catalog number: 22524‐1‐AP). Anti‐α‐synuclein antibody (D37A6), anti‐CHOP (L63F7) antibody and anti‐ubiquitin (P4D1) antibody were purchased from Cell Signaling Technology (Beverly, MA, USA). The secondary antibodies used in the study were as follows: donkey anti‐goat IgG H&L (Alexa Fluor^®^ 488) (ab150129), goat anti‐rabbit IgG H&L (Alexa Fluor^®^ 594) pre‐adsorbed (ab150084), goat anti‐mouse IgG H&L (HRP) (ab6789) and goat anti‐rabbit IgG H&L (HRP) (ab6721), which were all purchased from Abcam. PureProteome™ Protein A/G Mix Magnetic Beads was purchased from Millipore (Darmstadt, Germany).

The BCA protein assay kit was purchased from Thermo Fisher Scientific (NJ, USA). Acetonitrile, methanol and phosphonate acid were all HPLC grade reagents purchased from Thermo Fisher, JC‐1 and Mitochondrial Potential Sensors Kit (Invitrogen, Carlsbad, CA, USA).

### Cell culture and treatment

PC12 cells were cultured in RPMI‐1640 supplemented with 10% horse serum, 5% foetal bovine serum, 100 U/ml of penicillin and 100 μg/ml of streptomycin at 37°C under a humidified atmosphere containing 5% CO_2_. The cells were seeded into 96‐well plates at a density of 1 × 10^4^/well. To determine the non‐toxic dosages of ECH, PC12 cells were incubated with ECH (12.5–500 μM) for 24 hrs. To determine the toxic dosages of 6‐OHDA, PC12 cells were treated with various concentrations of 6‐OHDA (0–200 μM) for 24 hrs. To study the effect of ECH on cell viability in 6‐OHDA‐treated PC12 cells, after 80% confluence, the cells were pre‐incubated with different concentrations of ECH (0, 25, 50, 100 μM) in a serum‐free RPMI‐1640 medium for 1 hr. Subsequently, 6‐OHDA was added to the wells at a final concentration of 100 μM and incubated for another 24 hrs at 37°C.

### Transfection with siRNA

To study the effect of seipin inhibition, PC12 cells were transfected with seipin siRNA (Cat. No: 133000; Thermo Fisher Scientific). Briefly, a monolayer of 1 × 10^6^ cells in a 6‐well plate was transfected with 20 nM of siRNA using Lipofectamine2000™ (Life Technologies, Carlsbad, CA, USA). The cells were washed twice with phosphate‐buffered saline (PBS) to remove serum and supplemented with serum‐free medium containing a transfection cocktail. The transfected cells were incubated for 12 hrs followed by replacement of the growth medium containing the transfection cocktail with complete DMEM medium containing serum for an additional 10 hrs. The cells were then trypsinized, recounted and seeded at 2 × 10^5^ cells per well in 12‐well plates. The following day, cells were treated with or without 6‐OHDA to assess the silencing effect of specific targets on 6‐OHDA‐mediated ERS. The efficiency of gene silencing was assessed using Western blotting and found to be between 40% and 70%.

### Cell viability assay

Cell viability was determined using an MTT assay 22. A total of 0.5 mg/ml MTT was added to each well after the initial incubation, and incubated for further 4 hrs at 37°C. After the supernatant was removed, the formazan product was solubilized with 100 μl dimethyl sulfoxide (DMSO), and optical density at 570 nm was measured using an Epoch microplate assay reader (Bio‐Tek, Winooski, VT, USA).

### Transmission electron microscopy observation of PC12 cells

Following each group treatment described in Section 2.2, PC12 cells were washed with ice‐cold Ca^2+^‐free PBS, re‐suspended in PBS and centrifuged at 1000 r/min for 5 min. The cell precipitate was then fixed in 2.5% glutaraldehyde. After 90 min. of fixation, the cells were washed and post‐fixed in 1% OsO_4_ in a cacodylate buffer. The samples were then dehydrated with successively increasing concentrations of ethanol and embedded in Epon812. Thin sections were stained with uranyl acetate followed by lead citrate, and observed using a Hitachi HT‐7500 (Tokyo, Japan) transmission electron microscope.

### Measurement of intracellular ROS production using flow cytometry

Each group treatment described in ‘cell treatment’ section above washed with ice‐cold Ca^2+^ free PBS, and re‐suspended in PBS, centrifuged at 1000 r/min. for 5 min. The cells were centrifuged and washed twice with PBS. Cells were loaded with 10 μM H_2_DCFH‐DA for 30 min. at 37°C in the dark and analysed in a flow cytometer at 488 nm excitation.

### Animal surgical operation

All animal studies were performed with the approval of the Institutional Animal Care and Use Committee of Nanjing University of Chinese Medicine, and were performed according to the guidelines of the National Institutes of Health for the Care and Use of Laboratory Animals (NIH publication no. 80‑23). Male Sprague Dawley rats, weighing 200–220 g, were used in this study. Rat was housed individually in cages with food and water consumed *ad libitum*. Environmental conditions were strictly controlled, with a 12‐hr light/dark cycle, maintained at 24°C and 50% humidity.

Fifty animals were randomly divided into five groups, consisting of control (*n* = 10), vehicle (*n* = 10), 6‐OHDA (*n* = 10), and 6‐OHDA plus ECH‐high dosage (*n* = 10) and ‐low dosage (*n* = 10) groups. The control group did not undergo surgery. After anaesthesia with chloral hydrate (350 mg/kg), the skull was exposed and burr holes were drilled to introduce a syringe for injection (Fig. S1). The 6‐OHDA group and 6‐OHDA plus ECH group animals received 6‐OHDA (8 μg/4 μl in 0.9% saline containing 0.1% ascorbic acid) injections into the right substantia nigra pars compacta (SNpc; A/P:−5.0 mm, L:+ 1.9 mm, V: −7.8 mm) and ventral tegmental area (VTA; A/P: −4.6 mm, L: +0.9 mm, V: −7.5 mm) 23 with a 5‐μl Hamilton Syringe on stereotaxic apparatus (68002, RWD‐Life Science, Shenzhen, China). All injections were administered at a rate of 0.5 μl/min. The needle was left *in situ* for an additional 5 min. before retraction of the needle at a rate of 1.0 mm/min. Post‐surgery, with the skin sutured, the animals were removed from the stereotaxic instrument and kept under a heat lamp and a thermal blanket for 30 min. to maintain body temperature before being returned to their cage. Rats that showed positive results in a rotation test 3 weeks after surgery were selected as successful PD models. The vehicle group underwent the same procedures as the 6‐OHDA group except for receiving injections of saline instead of 6‐OHDA solution. Control, vehicle and 6‐OHDA groups were administered 0.9% saline (2 ml/kg) intraperitoneally for the following 14 days. The 6‐OHDA plus ECH‐high and ECH‐low dosage groups received ECH intraperitoneally at a dose of 7 and 3.5 mg/kg, respectively, for 14 days. The administration was carried out once a day at 8 o'clock a.m.

One day after the last injection, the rats were killed and the striatum and substantia nigra from five rats in each group were dissected and frozen in liquid nitrogen and stored at −80°C for analysis with high‐performance liquid chromatography coupled to tandem mass spectrometry (UPLC/MS/MS), Western blotting and reverse transcription‐polymerase chain reaction (RT‐PCR). Five animals per group were anaesthetized with chloral hydrate (350 mg/kg, i.p.) and perfused transcardially with 4% paraformaldehyde for pathological and immunohistochemical (IHC) studies. The whole procedure of the animal experiments is provided in the supplementary materials (Fig. S2).

### Rotation behavioural analysis

Rotation behavioural analysis was performed 3 and 5 weeks following surgery. Rats were injected with apomorphine (0.5 mg/kg, i.p.) and placed in a 40‐cm‐diameter stainless steel cylindrical bowl. Rotations over a 30‐min. period beginning 10 min. after the administration of apomorphine were counted. Data are expressed as contralateral net turns/min and results with the number of total turns over 210 were recorded and analysed. Assessments were carried out by an observer who was blind to the animal pre‐treatments.

### Determination of dopamine and metabolite concentrations

The striatum of the rats was rapidly dissected and stored at −80°C before the quantification of DA, DOPAC and HVA using UHPLC‐MS/MS. The experiments were performed using an Agilent 1290 ultra‐high‐performance liquid chromatography system consisting of a binary pump, autosampler and thermostated column compartment. The UPLC analyses were carried out using an elution profile composed of a first isocratic step of water (formic acid 0.1%), followed by acetonitrile (ACN) 95:5 for 0.5 min., and then 30% ACN for over 1 min. to separate DA, DOPAC and HVA at 25°C. The column was then washed with 90% ACN for 0.5 min., followed by equilibration of the column for 0.5 min. with 5% ACN. The flow rate was 0.3 ml/min. The injection volume was 5 μl. An Agilent 6460 triple quadrupole‐mass spectrometer with an electrospray ion source operated in the positive mode was used for detecting DA and DOPAC. The negative mode was used for detecting HVA. Flow injection analysis was used to optimize the fragmentor and source parameters. The optimized source parameters for MS analysis were as follows: drying gas temperature, 350°C; gas flow, 10 l/min.; nebulizer gas flow pressure, 35 PSI; and capillary voltage, 4500 V. The optimized fragmentor voltage, collision energy and MRM pairs are reported in Table 1.

**Table 1:** MRM chromatogram by UPLC‐MS/MS method for each substrate

Component	Parent ion	Daughter ion	Fragmentor (V)	Collision energy (V)	Polarity
DA	137	91.1	125	17	ESI+
DOPAC	123.1	77.2	140	20	ESI+
HVA	180.9	137	70	5	ESI‐

DA, DOPAC and HVA levels were calculated by extrapolating the peak area from a standard curve (ranging from 1 to 100 nM of mixed DA, DOPAC and HVA). The identification of peaks was carried out by comparison with standards. The amounts of DA, DOPAC and HVA in each sample were quantified by comparing the peak area of the samples to those of the standards. The units are expressed as ng/g of wet weight.

### Pathologic and immunohistochemical detection

After the rats were deeply anaesthetized and transcardially perfused, the brain was removed from the skull and dehydrated while immersed in the fixative solution for 1 week at 4°C. Slices of interest were selected from the SN‐VTA located −5.4 mm from bregma according to the atlas of Paxinos and Watson 23. Haematoxylin and eosin (HE) staining of paraffin‐embedded sections from each rat, with a slice thickness of 4 μm, was used to locate the SN‐VTA slices and observe neuronal morphology. The images were taken using an Olympus camera (DP72; Olympus, Tokyo, Japan) at an original magnification of 400×. Tissue sections were incubated with anti‐TH antibody (1: 200) in a blocking solution (Triton X‐100 0.4% v/v and NGS 3% v/v in PBS) for 1 h. After incubation, the slices were washed four times with PBS 0.01 M for 10 min each time. A second incubation with anti‐rabbit antibody (1:2000) was performed for 2 hrs at 25°C. After several washings with PBS, the antibody complex was detected using a modification of the ABC system (Kit Vectastain ABC, Vector Laboratory Inc., Burlingame, CA, USA). The midbrain dopaminergic cell groups were plotted from TH‐immunostained slices. TH‐immunoreactive (TH‐ir) neurons were stereologically quantified using Image‐Pro Express 6 (Media Cybernetics, Rockville, MD, USA). The mean number of TH‐ir neurons in each hemisphere, obtained through the quantification of four alternating slices, was considered to be representative of the SNpc neuronal cells in each animal. The selected areas were digitized with a microscope (DX45; Olympus). The images were taken using an Olympus camera (DP72; Olympus) at an original magnification of 100×.

For double immunofluorescence, the tissue sections were incubated with anti‐TH antibody and diluted (1:200) and anti‐BSCL2/Seipin antibody (1:200; Santa Cruz); TH was imaged with anti‐rabbit IgG H&L (Alexa Fluor^®^ 594) (1:200; Abcam) and seipin by anti‐goat IgG H&L (Alexa Fluor^®^ 488) (1:200; Abcam). The slides were mounted in a fluorescent mounting medium containing DAPI (Invitrogen). The sections were examined with a Zeiss Axiophot microscope equipped for epifluorescence illumination.

### Western blotting and ubiquitination detection

PC12 cells and striatum lysates were prepared in an ice‐cold lysis buffer (50 mM Tris‐HCl, 100 mM NaCl, 0.1% Triton X‐100, 0.1% SDS, 1 mM Na_3_VO_4_, 10 mM NaF and 1 mM EDTA) containing a 1% protease inhibitor cocktail (Sigma Chemical, St. Louis, MO, USA) and centrifuged at 16,000 *g* for 30 min. at 4°C. Next, 20 μg of total protein from each sample was subjected to SDS‐polyacrylamide gel electrophoresis (PAGE) (10%) and proteins were transferred to a PVDF membrane, which was subsequently incubated in 0.01 M PBS, 0.1% Tween‐20 and 5% non‐fat dry milk powder to block any remaining protein binding sites. Membranes were incubated overnight at 4°C using the following primary antibodies: the rabbit antibody against TH (1:2000), rabbit antibody against DAT (1:1500), mouse antibody against GAPDH (1:1000), rabbit antibody against seipin (BSCL2), GRP94, GRP78/Bip, ATF4, CHOP, alpha‐synuclein and ubiquitin antibody (1:1000). After three 5‐min. washes in TBST, membranes were incubated with the goat anti‐mouse or rabbit IgG (1:2000; Abcam) for 60 min. at room temperature. All antibody incubations and washing steps were carried out in 0.01 M PBS and 0.1% Tween‐20. The immunoreactive bands were visualized using an enhanced chemiluminescence detection system (ChemiDoc XRS+, Bio‐Rad, Hercules, CA, USA) and quantified with densitometry using ImageJ 1.49s software.

Detection of seipin ubiquitination followed by Yeun Su Choo's protocol 24 with little modification. Briefly, 1 × 10^9^ PC12 cells were harvested after treated with different conditions (6‐OHDA, ECH and 6‐OHDA plus ECH) were harvested and 1 ml complete cell lysis buffer of each group was collected to measure the protein concentration, to prepare Protein A/G Mix magnetic beads (Millipore) and anti‐seipin antibody (5 μg) conjunction according to the instructions and to incubate the prepared beads and 1 mg protein lysis at 2–8°C overnight to capture protein compounds cross‐linked with seipin. After immunoprecipitation and native elution, the samples were boil with 2 × SDS loading buffer, then load samples onto a SDS‐PAGE gel for immunoblotting analysis. Seipin ubiquitination was detected by anti‐ubiquitin antibody. Stripping membrane and staining Ponceau S was taken as loading control.

### Quantitative real‐time polymerase chain reaction

Frozen tissue samples were homogenized in 1 ml Trizol reagent, and total RNA was extracted following the manufacturer's protocol. After ethanol precipitation, the vacuum dried RNA was dissolved in 50 μl nuclease‐free water and the concentration and purity of isolated RNA was determined using Nanodrop 2000 (Thermo Fisher). The 260/280 nM ratios of the samples were between 1.7 and 2.1. The integrity of the RNA was assessed using an Agilent 2100 Bioanalyzer (Agilent, Palo Alto, CA, USA). Degradation was defined as a shift in the RNA size distribution towards smaller fragments and a decrease in fluorescence signal of ribosomal peaks. Only samples with defined ribosomal peaks were used in this study. For cDNA synthesis, 500 ng total RNA was mixed with 5 μl RT master mix (PrimeScript™ RT Master Mix, Otsu, Shiga, Japan). RNase‐free H_2_O was added to reach a final total volume of 10 μl. The solution was incubated for 15 min. at 37°C and terminated by incubating for 5 sec. at 85°C. Distilled water was added to the reaction mixture to reach a final volume of 40 μl. Real‐time qPCR was performed with an ABI 7500 Instrument (Bio‐Rad Laboratories) using the DNA binding dye SYBR Green I for the detection of PCR products. PCRs were set up in 96‐well plates using 2 μl cDNA with 10 μl 2× selected SYBR Green I master mix (life) containing forward and reverse PCR primers with RNase‐free H_2_O added to reach a final volume of 20 μl. The primer pairs used are listed in Table 2. Amplification was performed with a hot start polymerase activation step for 10 min. at 95°C, followed by 40 cycles of 15 sec. at 95°C and 1 min. at 60°C. The cycle threshold (CT) values of the target and reference genes were identified. All samples were run three times. The CT values were normalized by subtracting the CT value of the housekeeping gene GAPDH from the CT value of the target gene (ΔCT). The fold change between groups was calculated using the CT value calculated using the method of 2^−∆∆Ct^ (∆CT = CT [target gene] – CT [GAPDH]). Samples with an average CT value less than 32 were considered eligible for analysis. All samples were run three times.

**Table 2:** Primers used in real‐time polymerase chain reaction

Target gene	Sequence of primers	
Seipin	Forward	5′‐CCCACAAGTGATTTGAGTTGGGGA‐3′
	Reverse	5′‐GTGGCTGACGGTCGGCATGT‐3′
α‐synuclein	Forward	5′‐GGCGTCCTCTATGTAGGTTCC‐3′
	Reverse	5′‐GTGGGTACCCTTCTTCACCC‐3′
TH	Forward	5′‐CCCCACCTGGAGTATTTTGTG‐3′
	Reverse	5′‐ATCACGGGCGGACAGTAGACC‐3′
DAT	Forward	5′‐GTCACCAACGGTGGCATCTA‐3′
	Reverse	5′‐AATGCTGACCACGACCACAT‐3′
GRP94	Forward	5′‐TGCTGACCTTCGGGTTTGTG‐3′
	Reverse	5′‐TTCAGCTTGGAAGGCGAACT‐3′
GRP78/Bip	Forward	5′‐TACGAAGGTGAACGACCCCT‐3′
	Reverse	5′‐CAGGCGGTTTTGGTCATTGG‐3′
ATF4	Forward	5′‐CTACTAGGTACCGCCAGAAG‐3′
	Reverse	5′‐GCCTTACGGACCTCTTCTAT‐3′
CHOP	Forward	5′‐TGTTGAAGATGAGCGGGTGG‐3′
	Reverse	5′‐CCGGTTTCTGCTTTCAGGTG‐3′
GAPDH	Forward	5′‐GCATCTTCTTGTGCAGTGCC‐3′,
	Reverse	5′‐TACGGCCAAATCCGTTCACA‐3′

### Statistical analysis

SPSS Version 19.0 for Windows (IBM Corp, Armonk, NY, USA) was used for all statistical analyses. All experiments were performed at least three times. Comparisons between two groups were performed using a two‐samples Student's *t*‐test, and comparisons between multiple groups were performed using a one‐way ANOVA followed by Tukey's *post hoc* test. Data are expressed as mean ± S.D. *P *<* *0.05 was considered statistically significant.

## Results

### Protective effect of ECH on viability of PC12 cells injured by 6‐OHDA

In this study, we identified that ECH (chemical structure shown in Fig. 1A, CAS No. 82854‐37‐3) has a protective effect on nigrostriatal dopaminergic neurons. To determine non‐toxic dosages, we detected the cell viability of PC12 cells treated with different concentrations of ECH for 24 hrs, as shown in Figure 1B. Following treatment with concentrations of 12.5–200 μM of ECH for 24 hrs, no significant difference was seen in the viability of PC12 cells. However, with a concentration of 500 μM, cell viability was significantly lower than at other concentrations. Consequently, we chose to use concentrations of 25, 50 and 100 μM in the study.

We then examined PC12 cell viability following 6‐OHDA treatment. As shown in Figure 1C, 6‐OHDA reduced cell viability in a dose‐dependent manner and cytotoxicity was significantly induced at concentrations ≥100 μM 6‐OHDA (*P *<* *0.01). Therefore, we used 100 μM 6‐OHDA for 24 hrs as the optimal standard concentration and time course for the induction of cytotoxicity in the subsequent experiments.

To determine whether ECH exerted neuro‐protective effects against the action of 6‐OHDA, PC12 cells were pre‐incubated with the concentrations of 0, 25, 50 or 100 μM ECH for 1 hr followed by exposure to 100 μM 6‐OHDA for 24 hrs. The MTT assay showed that treating PC12 cells with 6‐OHDA alone resulted in a 20% reduction in the number of surviving cells after 24 hrs. Co‐treatment with 50 or 100 μM ECH showed a reduction in 6‐OHDA‐induced cytotoxicity (*P *<* *0.05 *versus* 6‐OHDA alone). PC12 cell viability increased in an ECH‐dose‐dependent manner compared with the cells treated with 6‐OHDA alone. This suggests that ECH reduced 6‐OHDA‐induced cytotoxicity.

### ECH protects against 6‐OHDA‐induced SNpc lesions *in vivo*

#### ECH maintained complete ultrastructure of endoplasmic reticulum damaged by 6‐OHDA *in vitro*

**Figure 2:**
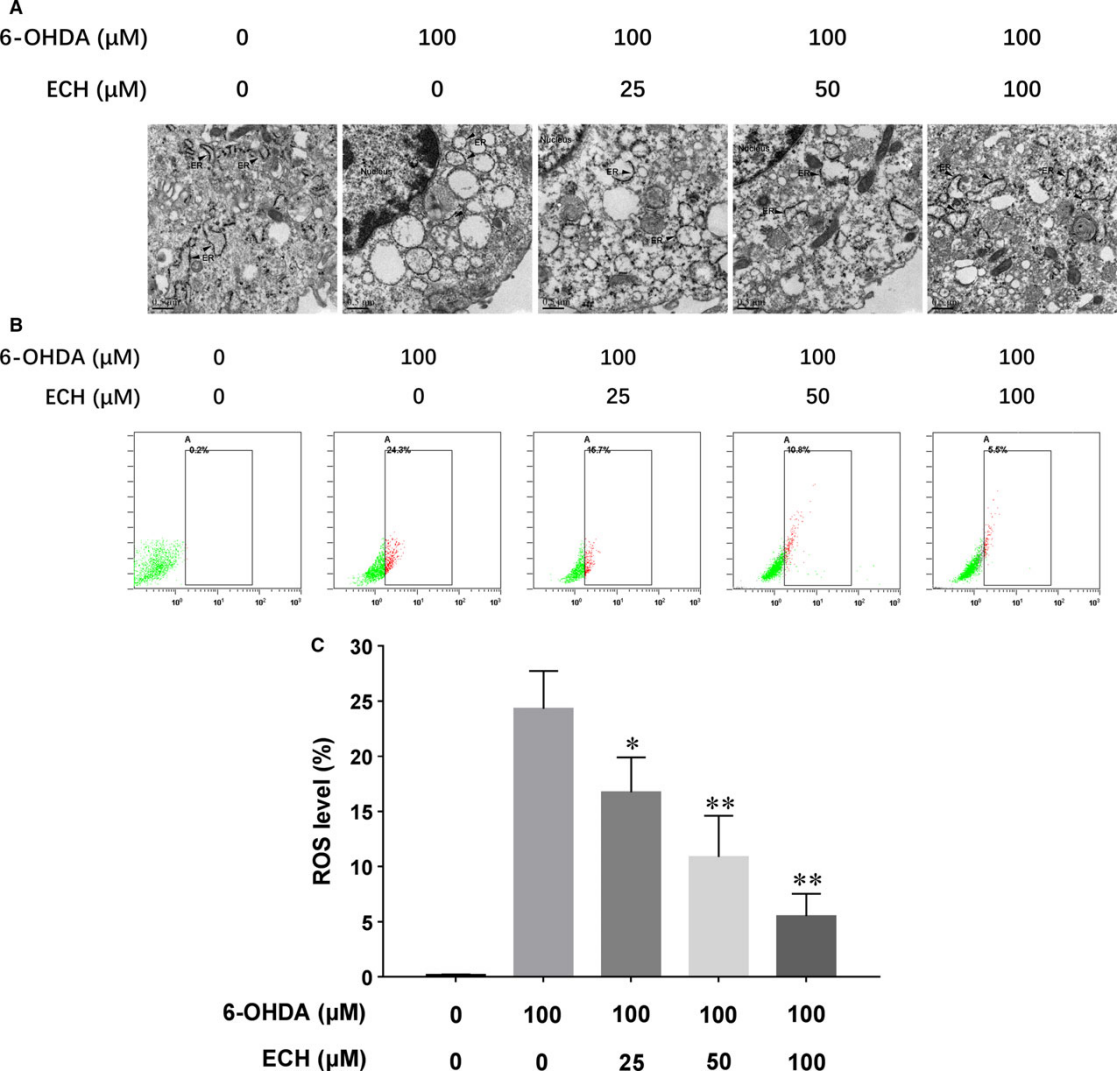
Ultrastructural evidence of ECH anti‐ERS (endoplasmic reticulum stress) effects as evaluated by transmission electron microscopy and ECH attenuated the 6‐OHDA‐induced intracellular ROS levels measured by flow cytometry. PC12 cells were pre‐incubated with different concentration of ECH (0, 25, 50, 100 μM) in a serum‐free RPMI‐1640 medium for 1 hr. Then, 6‐OHDA was added to the wells at a final concentration of 100 μM and incubated for another 24 hrs at 37°C. (**A**) 6‐OHDA‐induced ER curled and generated vacuole and ECH rescued ER structural integrity and made it extend. Black arrows, ER; scale bar, 0.5 μm. (**B**) ROS levels were determined using H2DCFH‐DA and measured by flow cytometry. (**C**) Data were calculated as ratio of fluorescence compared with untreated cells. Data are presented as mean ± S.D. values of three independent experiments. *n* = 3, *P <* *0.05, **P <* *0.01 *versus* 6‐OHDA‐treated PC12 cells.

To evaluate ER morphology transformation following 6‐OHDA and ECH treatment, the ER ultrastructure was determined using transmission electron microscopy. The results show that 100 μM 6‐OHDA‐induced ER curling and generated a vacuole, shown in Figure 2A. The ECH‐treated group showed recovery of ER structural integrity and caused extension of the ER. The ER is marked by a black arrow.

#### ECH attenuated intracellular ROS production induced by 6‐OHDA *in vitro*

As shown in Figure 2B, cells treated with 6‐OHDA showed a significant increase (approximately 1.4‐fold) of intracellular ROS production compared with untreated cells (*P *<* *0.01). This increase was significantly attenuated by co‐treatment with 25, 50 or 100 μM ECH, shown in Figure 2C (*n* = 3, *P <* *0.05, **P <* *0.01).

### ECH protects against 6‐OHDA‐induced SNpc lesions *in vivo*

#### ECH reduced pathologic lesions and up‐regulated tyrosine hydroxylase (TH) expression in SNpc following 6‐OHDA treatment

**Figure 3:**
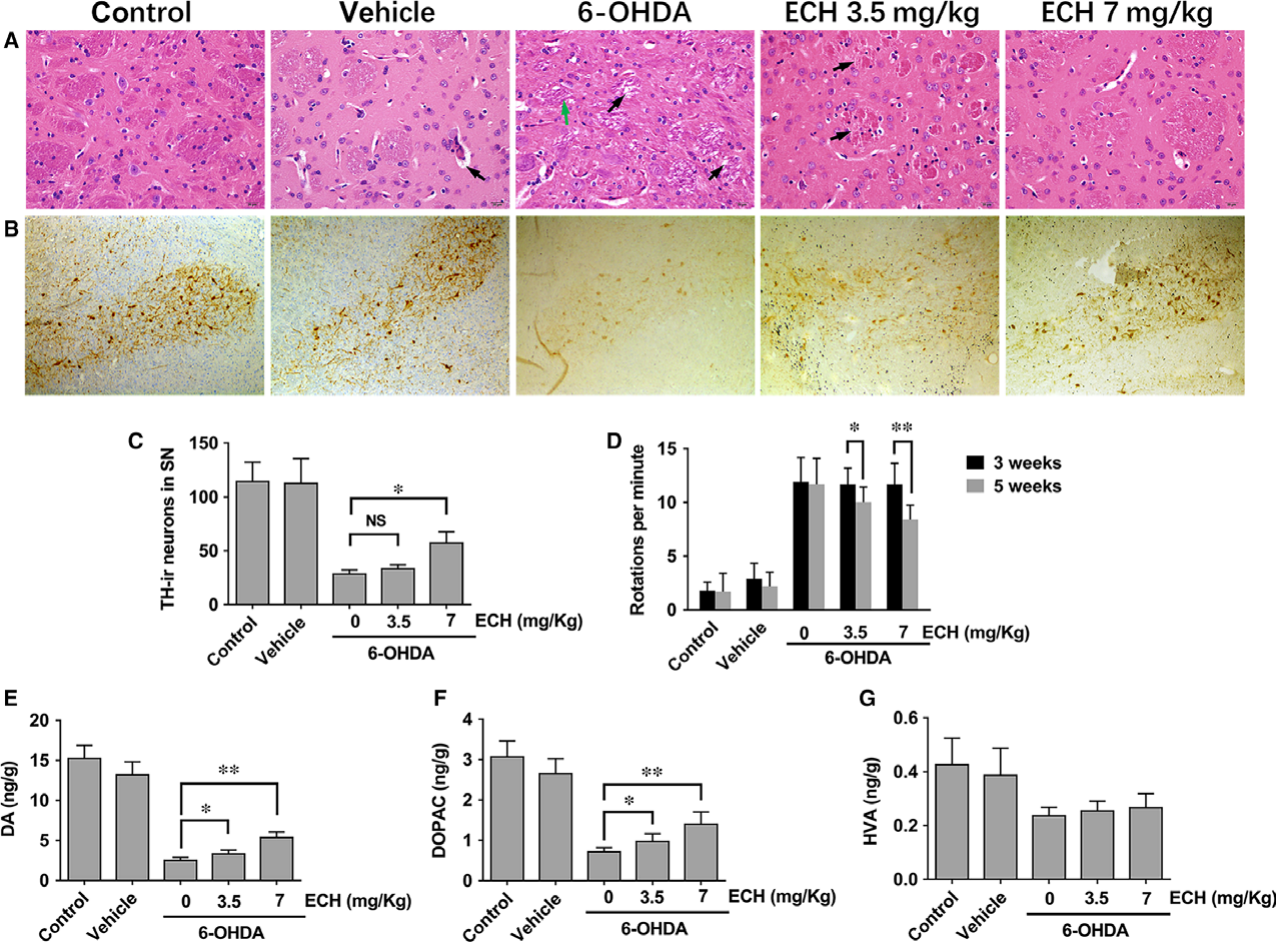
Nigrostriatal dopaminergic protection of ECH in 6‐OHDA‐induced rat. (**A**) ECH maintained neuronal morphology in striatum: vehicle group, black arrow, angioedema, caused by the surgery; 6‐OHDA group, green arrow, disordered fibre cord, black arrow, white matter oedema; ECH low concentration group, black arrow, white matter oedema; ECH‐high concentration group, no obvious oedema. (**B**) ECH rescued the number of tyrosine hydroxylase(TH)‐immunoreactive DA cells in the substantia nigra (SN) induced by 6‐OHDA damaged. Immunohistochemistry assay of TH taken in 4‐μm paraffin section of the SN. Brown region, TH‐immunoreactive DA cell bodies (20 × objective under white light). (**C**) Relative quantitative counting of TH‐immunoreactive DA cells in each group. The control group taken as 100 per cent compared with other groups (*n* = 5, NS, No significant, *P <* *0.05). (**D**) ECH protected effects on apomorphine‐induced rotational behaviours: average rotational turns per min are shown at different time‐points (*n* = 10). Data are expressed as the number of net rotations per minute (mean ± S.D.) over a 30‐min. period following apomorphine administration at 3 and 5 weeks after the lesion. *P <* *0.05, **P <* *0.01. (**E–G**) Effects of ECH on dopamine and metabolite concentrations in striatum: DA (**E**), DOPAC (**F**) and HVA concentrations in the striatum of each group (*n* = 5, *P <* *0.05, **P <* *0.01). (**G**)The HVA concentration of the control and vehicle group was higher than that other group (*P *<* *0.01), but no significant statistical difference among 0, 3.5, 7 mg/kg ECH groups.

To verify the neurotoxic effects of 6‐OHDA and the protective effects of ECH, we observed pathological changes in the striatum using HE staining. The main morphological changes in the 6‐OHDA‐treated rat model in this study were characterized by white matter oedema in the striatum, enlargement of nerve fibres, and neuronal degeneration and necrosis in the SN. As showed in Figure 3A, abnormalities such as nerve cell degeneration, necrosis and white matter oedema, congestion in interstitial vasculature, infiltration of inflammatory cell and proliferation of glial cells were not observed in the control or vehicle groups. In the 6‐OHDA and 6‐OHDA plus ECH groups, white matter oedema in the striatum was seen. In the SNpc, part of the nerve cell structure was not clear, the nucleus was no longer apparent, and the cytoplasm had a granular appearance. The nuclei of individual cells were deeply stained and the size of the nuclei was reduced. However, in the 6‐OHDA plus high‐dose ECH group, there was a significant reduction in the extent of these changes compared with the 6‐OHDA group.

We examined the extent of 6‐OHDA‐induced dopaminergic neuronal degeneration in the SN by TH immunohistochemistry. As showed in Figure 3B, in the control vehicle groups, dopaminergic neurons in the SN were intensely immunoreactive to TH (TH‐ir) and there was no significant difference in the number of TH‐ir neurons. The number of TH‐ir neurons in the unlesioned side was not significantly different between groups (data not shown here). However, compared with the control and vehicle groups, the number of TH‐ir neurons in the lesioned side of 6‑OHDA‐treated rats was significantly reduced. Compared with the 6‐OHDA group, the number of TH‐ir neurons in the 6‐OHDA plus high‐dose ECH group was significantly higher (Fig. 3C, *n* = 5, *P *<* *0.05).

#### ECH decreased the effect of 6‐OHDA on apomorphine‐induced rotations *in vivo*

Apomorphine‐induced rotation tests were performed 3 and 5 weeks after the surgery. Rats were injected with vehicle (sham group), 6‐OHDA and/or 6‐OHDA plus ECH at a dose of 3.5 or 7 mg/kg. The number of rotations was recorded at the indicated time‐points. As shown in Figure 3D, 3 weeks post‐lesion, unilateral lesioned rats injected with 6‐OHDA showed a trend of increased contralateral rotation after apomorphine administration compared with the vehicle group (*P *< 0.01), but there was no significant difference between the 6‐OHDA group and the 6‐OHDA plus ECH group (*P* > 0.05). At 5 weeks post‐lesion, the 6‐OHDA plus ECH at 3.5 mg/kg (*P *<* *0.05) and 7 mg/kg (*P *<* *0.01) group showed a significantly decreased number of rotations. The typical rotation behaviour of rats was shown in the supplementary video (Video S1).

#### ECH rescued dopamine and metabolite concentrations in the striatum following 6‐OHDA treatment *in vivo*

As shown in Figure 3E–G, DA and metabolite levels in the striatum were determined using UHPLC‐MS/MS. DA, DOPAC and HVA concentrations in the striatum of the 6‐OHDA group and the 6‐OHDA plus ECH group were significantly lower than that in the control and vehicle groups (*P < *0.01). DA and DOPAC concentrations in the 6‐OHDA plus ECH group were higher than those in the 6‐OHDA group (Fig. 3E and F, *P* < 0.05 or *P *<* *0.01). Furthermore, the HVA concentration in the control and vehicle group was higher than that in the other three groups (*P *<* *0.01). However, there was no statistically significant difference between the 6‐OHDA plus ECH 0, 3.5, 7 mg/ml groups (Fig. 3G).

### ECH rescues protein and mRNA expression levels of TH and DAT, and decreases α‐synuclein accumulation in 6‐OHDA‐treated rats

**Figure 4:**
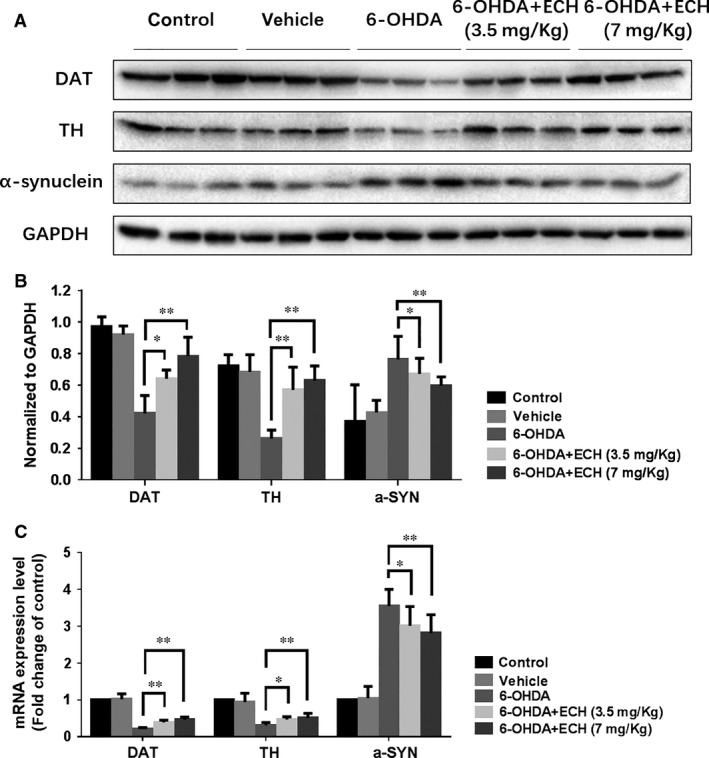
ECH rescued protein and mRNA expression levels of TH, DAT and decreased α‐synuclein accumulation in 6‐OHDA‐induced rat. (**A**) After intraperitoneally treated with vehicle or 0, 3.5, 7 mg/kg of ECH for 14 days, relative protein levels were measured using Western blotting (*n* = 3), GAPDH taken as a reference gene. (**B**) The immunoreactive bands were quantified by densitometry using ImageJ software and normalized to GAPDH (*n* = 3, *P <* *0.05, **P <* *0.01). (**C**) The relative mRNA levels of DAT, TH and α‐synuclein were determined using RT‐PCR. The results of RT‐PCR were normalized to GAPDH, and expressed as fold change to control. (The values represent the mean ± S.D. of triplicate experiments, *P <* *0.05, **P <* *0.01).

To determine the neurotoxicity of 6‐OHDA and protective effect of ECH, we studied the protein and mRNA expression levels of TH, DAT and α‐synuclein in the striatum of rats. As shown in Figure 4, treatment with 6‐OHDA resulted in increased protein and mRNA expression levels of α‐synuclein, indicating its accumulation. Decreased protein and mRNA expression levels of TH and DAT indicated the apoptosis of dopaminergic neurons. Compared with the 6‐OHDA group, the 6‐OHDA + ECH groups had significantly increased TH and DAT mRNA and protein expression, and reduced α‐synuclein accumulation (*n* = 3, *P *<* *0.05).

ECH protects dopaminergic neurons from 6‐OHDA‐induced TH down‐regulation and seipin overexpression *in vivo* and inhibits ERS‐associated protein Bip expression through seipin *in vitro*.

#### Effect of ECH on co‐localization of TH and seipin in 6‐OHDA‐treated rats

**Figure 5:**
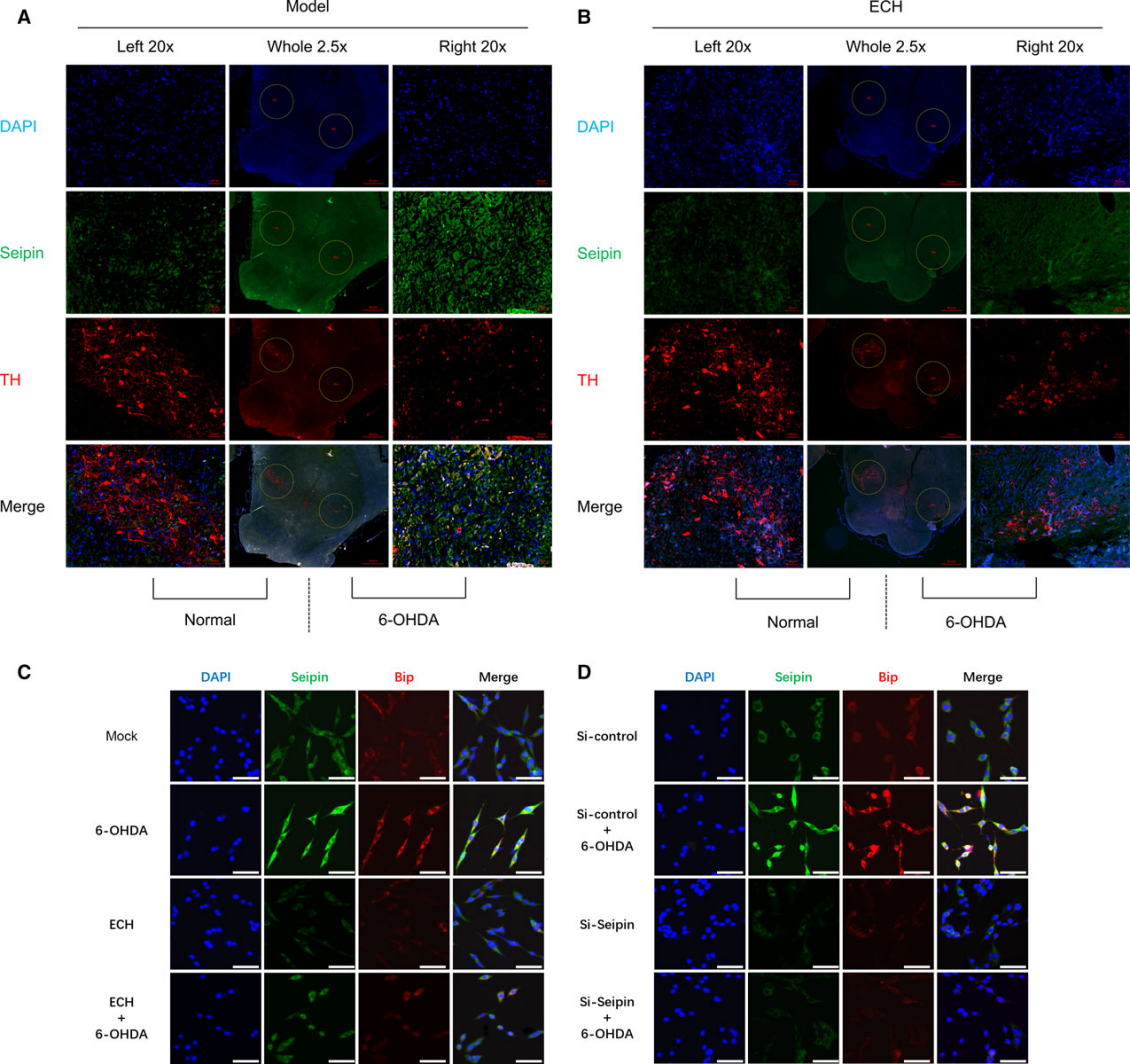
ECH protected Nigrostriatal neurons from 6‐OHDA‐induced TH downregulation and seipin overexpression *in vivo* and inhibited ERS‐associated protein Bip expression through seipin *in vitro*. (**A** and **B**) Immunofluorescence assay about brain SNpc slices in the model group and 7 mg/kg/day ECH group. Left, untreated side, Right, operation lesion side, Whole, the whole brain(2.5 × objective). The circle showed SNpc area of each side (20 × objective); (**A**) 6‐OHDA caused seipin accumulation and TH expression inhibited in SNpc. (**B**) ECH inhibited seipin and Bip overexpression induced by 6‐OHDA in SNpc. Blue, DAPI; green, Seipin; red, TH; scale bar, 50 μM. (**C** and **D**) Immunofluorescence assay about PC12 cells treated with different conditions. (**C**) ECH attenuated seipin and Bip expression in cytoplasm. (**D**) Modified seipin expression by siRNA could inhibit Bip overexpression induced by 6‐OHDA. Blue, DAPI; green, Seipin; red, Bip; scale bar, 50 μM.

As the neurotoxicity of 6‐OHDA and protective effect of ECH in striatum were verified, we wondered if the protective effect occurring in the SNpc was associated with the reduction of seipin accumulation. To validate this hypothesis, tissue sections from the 6‐OHDA group (Fig. 5A, model) and 6‐OHDA + ECH (7 mg/kg) group (Fig. 5B, ECH) were stained for TH (red) and seipin (green), and the nuclei were counterstained with DAPI (blue). The sections were examined with a Zeiss Axiophot microscope equipped for epifluorescence illumination. Different channels were merged to obtain co‐localization of seipin with the DA neuromarker, TH. As shown in Figure 5A, in the same rat's brain, after three‐dimensional stereotactic injection of 6‐OHDA, the number of TH‐positive neurons in the SNpc was reduced and seipin expression increased on the lesioned side. In the ECH group, TH‐positive neurons on the lesioned side were rescued from 6‐OHDA neurotoxicity and the accumulation of seipin induced by 6‐OHDA was decreased (Fig. 5B).

#### ECH inhibited ERS‐associated protein Bip expression through seipin *in vitro*

According to the results in Figure 2, we verified that 6‐OHDA could induce ER damage and ECH could attenuate this effect in PC12 cells. As shown in Figure 5A, 6‐OHDA treatment caused damage to dopaminergic neurons and seipin overaccumulation. Therefore, we speculated that seipin overexpression might be related to ERS and could be regulated by ECH. In Figure 5C, the results demonstrated that seipin and the ERS‐associated protein, Bip, were up‐regulated by 6‐OHDA administration, and that this accumulation was attenuated by ECH treatment. In Figure 5D, Bip accumulation induced by 6‐OHDA was reversed by administration of the siRNA for seipin. These results indicated that ECH could decrease seipin overexpression induced by 6‐OHDA and that seipin accumulation was a crucial factor leading to ERS.

**Figure 6:**
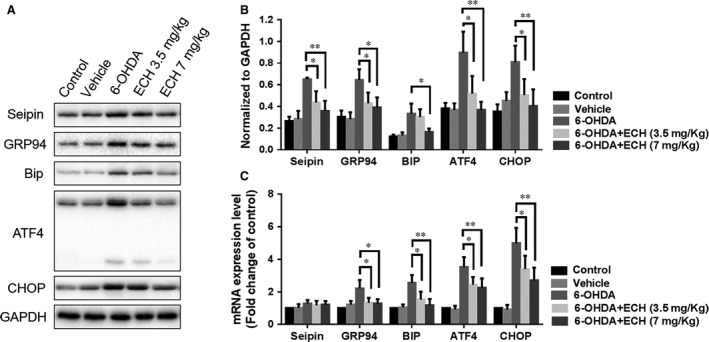
ECH protected Nigrostriatal neurons from 6‐OHDA‐induced ERS‐associated proteins upregulation and inhibited seipin over accumulation *in vivo*. (**A**) Protein lysis solution of 5 rats’ striatum in each group pooled together. Seipin, GRP94, Bip, ATF‐4, CHOP, GAPDH protein expression levels detected by Western blotting in groups treated with different conditions (*n* = 5, 40 μg protein each lane). (**B**) The immunoreactive bands were quantified by densitometry using ImageJ software and normalized to GAPDH. (*n* = 3, *P <* *0.05, **P <* *0.01). (**C**) The relative mRNA levels of seipin, GRP94, Bip, ATF‐4, CHOP were determined using RT‐PCR. The results of RT‐PCR were normalized to GAPDH and expressed as fold change to control. Seipin mRNA level among each group had no significant difference. But ERS‐associated gene transcription had obvious significant. (*n* = 6, the values represent the mean ± S.D. of triplicate experiments, *P <* *0.05, **P <* *0.01).

### ECH protects dopaminergic neurons from 6‐OHDA‐induced ERS‐associated protein up regulation and inhibits seipin over accumulation *in vivo*

To further confirm the role of ECH in the ERS pathway and regulation of seipin expression *in vivo*, we studied protein and mRNA expression levels of seipin, GRP94, GRP78/BIP, ATF4 and CHOP in the striatum of rats. As shown in Figure 6, treatment with 6‐OHDA resulted in increased protein (*n* = 5, rats’ striata pooled to reach a sufficient protein concentration, *P *<* *0.05, Fig. 6A and B) and mRNA (*n* = 6) expression levels of GRP94, BIP, ATF4 and CHOP (Fig. 6C). Interestingly, the mRNA level of seipin was not significantly increased following 6‐OHDA or ECH treatment, while the protein expression level of seipin could be affected by 6‐OHDA and ECH. Therefore, we speculated that the accumulation or degradation of seipin was due to post‐transcriptional regulation. 6‐OHDA is known to activate various ERS markers and chaperones, and initiate their downstream cascades leading to an increase in CHOP, activating apoptosis. Compared with the 6‐OHDA group, the 6‐OHDA + ECH groups had significantly decreased expression of seipin, GRP94, BIP, ATF4 and CHOP (*P *<* *0.05 or *P *<* *0.01), which may explain how ECH protects dopaminergic neurons.

### ECH protects against 6‐OHDA‐induced ERS through promoting seipin ubiquitination and degradation

**Figure 7:**
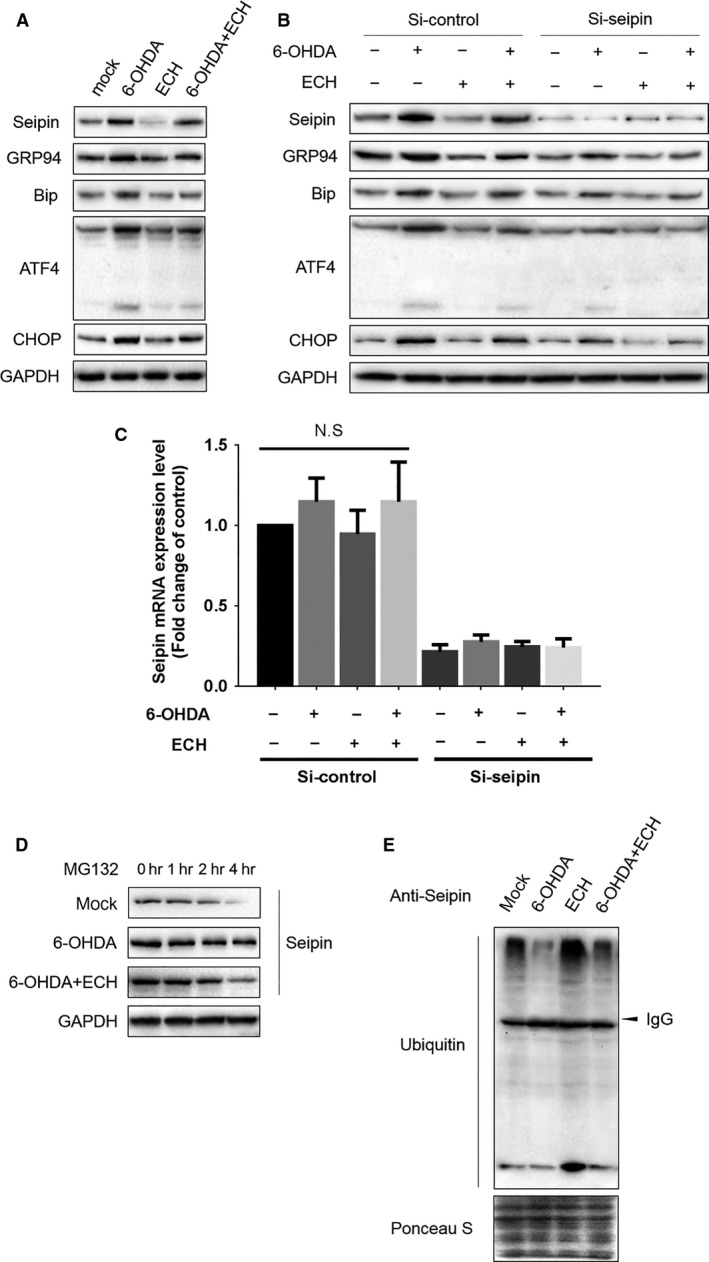
ECH protected PC12 from 6‐OHDA‐induced ERS through promoting seipin ubiquitination and degradation. (**A**) ECH down‐regulated seipin and ERS‐associated protein expression induced by 6‐OHDA. Seipin, GRP94, Bip, ATF‐4, CHOP, GAPDH protein expression levels detected by Western blotting in groups treated with different conditions (*n* = 3, 40 μg protein each lane). (**B**) Knocking down seipin by siRNA attenuated ERS‐associated protein accumulation induced by 6‐OHDA. Seipin, GRP94, Bip, ATF‐4, CHOP, GAPDH protein expression levels detected by Western blotting in groups treated with different conditions (*n* = 3, 40 μg protein each lane). SiRNA, 50 nM each group transfected and collected to obtain protein lysis after 48 hrs. (**C**) The relative mRNA levels of seipin were determined using RT‐PCR. The results of RT‐PCR were normalized to GAPDH and expressed as fold change to control. SiRNA of seipin dramatically inhibited mRNA levels of seipin (*P *<* *0.01), but there was no significant difference among each group transfected by Si‐control (*n* = 3, the values represent the mean ± S.D. of triplicate experiments). (**D**) ECH promoted seipin degradation under 6‐OHDA treatment. MG132, 50 μM; lane1‐lane4, 0, 1, 2, 4 hrs, *n* = 3, 40 μg protein each lane. (**E**) 6‐OHDA blocked seipin ubiquitination but ECH rescued it. Protein lysis of each group was purified and pulled down by anti‐seipin antibody and detected by anti‐ubiquitin antibody. Ponceau S staining of the whole PVDF membrane containing all samples taken as loading control.

To validate the hypothesis of ECH regulation of the ERS pathway through seipin, we knocked down seipin in PC12 cells using siRNA and measured ERS‐associated protein expression. As shown in Figure 7A, the protein expression levels of seipin, GRP94, BIP, ATF‐4 and CHOP were up‐regulated by 6‐OHDA and reversed by ECH. When seipin expression level was knocked down using siRNA, up‐regulation of proteins in the ERS pathway induced by 6‐OHDA was attenuated and further dramatically blocked by ECH (Fig. 7B). Furthermore, the results in Figure 7C indicated that the mRNA expression level of seipin was inhibited by siRNA but was not influenced by ECH or 6‐OHDA. To determine whether ECH played a crucial role in seipin post‐transcriptional regulation, seipin degradation was analysed in PC12 cells under MG132 treatment. As shown in Figure 7D, under MG132 treatment, 6‐OHDA inhibited the degradation of seipin; however, ECH could accelerate this process. Therefore, we speculated the rescue degradation of seipin was relayed on ubiquitin‐activating enzyme. More evidence of ECH promoting the ubiquitination of seipin was shown in Figure 7E; 6‐OHDA interrupted the combination of ubiquitin and seipin, while ECH promoted the formation of seipin‐ubiquitin compounds. These results demonstrate that ECH protects against 6‐OHDA‐induced ERS through promoting seipin ubiquitination and degradation.

## Discussion

PD is a common and complex neurological disorder. It is now viewed as a slowly progressive neurodegenerative disorder that begins years before a diagnosis can be made, implicates multiple neuroanatomical areas, results from a combination of genetic and environmental factors, and manifests with a broad range of symptoms. The gold standard for the diagnosis of PD has been the presence of SNpc degeneration and Lewy pathology at post‐mortem pathological examination. A major goal of PD research is the development of disease‐modifying drugs that slow or stop the underlying neurodegenerative process. However, the association between Lewy pathology and pathogenesis of the disease is poorly understood 25. In recent years, an increasing number of studies have aimed to identify more effective strategies to target selected dysfunctional molecular pathways in specific patients and to target several molecular pathways. Several intracellular signalling pathways have been shown to be involved in neuronal damage during PD. Besides ERS, mitogen‐activated protein kinases (MAPKs), including extracellular signal‐regulated kinase 1/2 (ERK1/2), p38 and c‐Jun N‐terminal kinase (JNK), are usually involved in neuronal cell apoptosis and death 26. The phosphoinositide 3‐kinase (PI3K)/AKT pathway is also an important contributor in retarding the dopaminergic cell death process 27.

The ER plays a very important role in protein folding in eukaryote cells and any perturbations altering ER homoeostasis can trigger the disruption of the folding process and accumulation of misfolded or unfolded proteins. ERS usually induces UPR as a physiological protective response aimed at restoring ER homoeostasis and normal cell function if it is transiently induced 28. ERS and activation of UPR are closely involved in the pathophysiology of PD 13, 29. A mild ERS (‘preconditioning’) is neuroprotective in *Drosophila* and mouse models of Parkinson disease 30. However, under chronic ERS, the accumulation of unfolded proteins and sustained UPR activity triggers the activation of pro‐apoptotic pathways and cell death, thereby eliminating damaged cells 31. Meanwhile, chronic ERS could contribute to neurodegeneration in α‐synucleinopathy 32. In this study, we verified that the mRNA and protein expression of GRP94, BIP, ATF4 and CHOP increased in both *in vivo* and *in vitro* studies, indicating that the Grp94/Bip‐ATF4‐CHOP signal pathway is closely associated with the pathology and pathogenesis of PD.

Seipin/BSCL2 was first identified as a causative gene of BSCL2, the most severe form of lipodystrophy in human beings, characterized by a near complete lack of adipose tissue and subsequent metabolic disturbance 33. Furthermore, it is a ubiquitously expressed oligomeric ER transmembrane protein 34. Seipin is an integral membrane protein of the endoplasmic reticulum (ER) with two predicted transmembrane domains, an intraluminal loop, and amino‐ and carboxyterminal intracytoplasmic ends 35. Seipin has a key role in adipogenesis, lipid droplet homoeostasis and cellular triglyceride lipolysis. Seipin‐deficiency strongly impairs adipocyte homoeostasis and leads to lipodystrophy 36, while others have shown a potential neural involvement 15, 37, 38, 39. Several have validated that seipin is a key molecule regulating neuronal diseases such as PD 40, AD 41, Celia's Encephalopathy 42 and others 43, 44. Several lines of evidence that UPR and ERS play an important role in the pathogenesis of seipin‐related motor neuron diseases 15, 43. Most studies have focused on seipin mutation leading to misfolded protein aggregation in the ER, triggering ERS 15, 45. Mutations in the N‐glycosylation site of seipin are associated with the disease state and result in accumulation of unfolded proteins in the ER, leading to the UPR and cell death, suggesting that these mutations leading to disease are tightly associated with ERS 38, 45. However, Guo *et al*. showed that both N88S/S90L‐mutant and wild‐type seipin knocked‐in mice presented with mild ERS, but autophagosomes were significantly increased in mutant mice 46. We found that a 6‐OHDA‐induced rat model also presented with seipin aggregation, leading to ERS; 6‐OHDA exposure induced a marked activation of GRP78 and CHOP expression, indicative of ERS, which has been reported in many previous studies 47, 48. We also found that the accumulation of seipin (BSCL2) was significantly attenuated by ECH and ECH blocked the aggregation of seipin induced by 6‐OHDA through promoting its ubiquitination and degradation. Furthermore, attenuation of seipin accumulation dramatically reduced activation of ERS‐related pathways.

In recent years, ECH was valued as one of the new potential medicines for the treatment of PD because of its many neuroprotective effects. Several studies have shown that it significantly improves motor behaviour and suppresses the loss of nigral DA neurons in MPTP‐lesioned mice 49. Other studies have shown that ECH protects neurons through the regulation of the ROS/ATF3/CHOP pathway 50 or regulating the stress‐active p38MAPK and NF‐κB p52 signals 51. The present study revealed for the first time that ECH has a protective effect against 6‐OHDA‐induced ERS in nigrostriatal dopaminergic neurons through reducing the accumulation of seipin *in vivo* and *in vitro*.

ECH has been proven to possess a variety of pharmacological activities; however, it was also reported that the bioavailability of ECH in rats is very low 52. How ECH plays a role in nerve protection remains unknown. However, according to Jun Zhou's research, the significantly higher plasma concentrations and lower CL of ECH were found in PD rats. The ECH kinetics in PD model rats differed from those in normal rats, perhaps due to the physiological changes induced by PD 53. Meanwhile, scientists have also been searching for some effective methods to address this issue. It was reported that ECH oral bioavailability was elevated by verapamil and clove oil in an animal model by nearly 1.37‐fold and 2.36‐fold, respectively, when compared with ECH alone 54. The dietary and medicinal *C. tubulosa* extract can enhance the intestinal absorption of ECH 55. We are looking forward to more pharmacokinetic studies of ECH in the future.

There are several limitations in our work. First, although we demonstrated that ECH had an obvious neuroprotective function through acting on seipin, we did not determine the precise mechanism of how seipin aggregated under 6‐OHDA treatment and regulated ERS. Second, our evidence was obtained in either PC12 cells or striatal neurons from Sprague Dawley rats, and the real function of ECH on the patients with PD may deserve further investigation. In summary, ECH could be a novel approach in PD therapy.

## Funding source

This work was supported by Nanjing Science and Technology Development Project (Grants No. 201605036). Nanjing science and Technology Committee, Nanjing, Jiangsu, China.

## Conflict of interests

The authors declare no conflict of interests.

## Supporting information


**Figure S1**. Operative procedure and injection on the rat model. (a) vertical view (b) lateral view.
**Figure S2**. The whole procedure of animal experiments.

**Video S1.** The typical rotation behavior of rats.MOV 68.6 MB.

## References

[1] Noelker C , Bacher M , Gocke P , *et al* The flavanoide caffeic acid phenethyl ester blocks 6‐hydroxydopamine‐induced neurotoxicity. Neurosci Lett. 2005; 383: 39–43. 15894425 10.1016/j.neulet.2005.04.023

[2] Wirdefeldt K , Adami HO , Cole P , *et al* Epidemiology and etiology of Parkinson's disease: a review of the evidence. Eur J Epidemiol. 2011; 26: S1–58. 21626386 10.1007/s10654-011-9581-6

[3] Lees AJ , Hardy J , Revesz T . Parkinson's disease. Lancet. 2009; 373: 2055–66. 19524782 10.1016/S0140-6736(09)60492-X

[4] Devic I , Hwang H , Edgar JS , *et al* Salivary alpha‐synuclein and DJ‐1: potential biomarkers for Parkinson's disease. Brain. 2011; 134: e178. 21349902 10.1093/brain/awr015 PMC3122368

[5] Chen CM , Liu JL , Wu YR , *et al* Increased oxidative damage in peripheral blood correlates with severity of Parkinson's disease. Neurobiol Dis. 2009; 33: 429–35. 19110057 10.1016/j.nbd.2008.11.011

[6] Garcia‐Moreno JM , Martin de Pablos A , Garcia‐Sanchez MI , *et al* May serum levels of advanced oxidized protein products serve as a prognostic marker of disease duration in patients with idiopathic Parkinson's disease? Antioxid Redox Signal. 2013; 18: 1296–302. 23121480 10.1089/ars.2012.5026

[7] Bruggink KA , Kuiperij HB , Ekholm‐Pettersson F , *et al* Detection of elevated levels of alpha‐synuclein oligomers in CSF from patients with Parkinson disease. Neurology. 2011; 77: 510–1. 21810701 10.1212/WNL.0b013e318219dd92

[8] Mollenhauer B , Locascio JJ , Schulz‐Schaeffer W , *et al* alpha‐Synuclein and tau concentrations in cerebrospinal fluid of patients presenting with parkinsonism: a cohort study. Lancet Neurol. 2011; 10: 230–40. 21317042 10.1016/S1474-4422(11)70014-X

[9] Sala G , Stefanoni G , Arosio A , *et al* Reduced expression of the chaperone‐mediated autophagy carrier hsc70 protein in lymphomonocytes of patients with Parkinson's disease. Brain Res. 2014; 1546: 46–52. 24361989 10.1016/j.brainres.2013.12.017

[10] Parnetti L , Chiasserini D , Persichetti E , *et al* Cerebrospinal fluid lysosomal enzymes and alpha‐synuclein in Parkinson's disease. Movement Disord. 2014; 29: 1019–27. 24436092 10.1002/mds.25772 PMC4282452

[11] Mondello S , Constantinescu R , Zetterberg H , *et al* CSF alpha‐synuclein and UCH‐L1 levels in Parkinson's disease and atypical parkinsonian disorders. Parkinsonism Relat Disord. 2014; 20: 382–7. 24507721 10.1016/j.parkreldis.2014.01.011

[12] Song IU , Chung SW , Kim JS , *et al* Association between high‐sensitivity C‐reactive protein and risk of early idiopathic Parkinson's disease. Neurol Sci. 2011; 32: 31–4. 20532580 10.1007/s10072-010-0335-0

[13] Roussel BD , Kruppa AJ , Miranda E , *et al* Endoplasmic reticulum dysfunction in neurological disease. Lancet Neurol. 2013; 12: 105–18. 23237905 10.1016/S1474-4422(12)70238-7

[14] Kraskiewicz H , FitzGerald U . InterfERing with endoplasmic reticulum stress. Trends Pharmacol Sci. 2012; 33: 53–63. 22112465 10.1016/j.tips.2011.10.002

[15] Ito D , Suzuki N . Molecular pathogenesis of seipin/BSCL2‐related motor neuron diseases. Ann Neurol. 2007; 61: 237–50. 17387721 10.1002/ana.21070

[16] Ito D , Suzuki N . Seipinopathy: a novel endoplasmic reticulum stress‐associated disease. Brain. 2009; 132: 8–15. 18790819 10.1093/brain/awn216

[17] Lei L , Yang F , Zhang T , *et al* Preparative isolation and purification of acteoside and 2’‐acetyl acteoside from Cistanches salsa (C.A. Mey.) G. Beck by high‐speed counter‐current chromatography. J Chromatogr A. 2001; 912: 181–5. 11307982 10.1016/s0021-9673(01)00583-0

[18] Wang YH , Xuan ZH , Tian S , *et al* Echinacoside Protects against 6‐Hydroxydopamine‐Induced Mitochondrial Dysfunction and Inflammatory Responses in PC12 Cells *via* Reducing ROS Production. Evid Based Complement Alternat Med. 2015; 2015: 189239. 25788961 10.1155/2015/189239 PMC4348598

[19] Ungerstedt U . 6‐Hydroxy‐dopamine induced degeneration of central monoamine neurons. Eur J Pharmacol. 1968; 5: 107–10. 5718510 10.1016/0014-2999(68)90164-7

[20] Cohen G , Heikkila RE . The generation of hydrogen peroxide, superoxide radical, and hydroxyl radical by 6‐hydroxydopamine, dialuric acid, and related cytotoxic agents. J Biol Chem. 1974; 249: 2447–52. 4362682

[21] Rodriguez‐Blanco J , Martin V , Herrera F , *et al* Intracellular signaling pathways involved in post‐mitotic dopaminergic PC12 cell death induced by 6‐hydroxydopamine. J Neurochem. 2008; 107: 127–40. 18665912 10.1111/j.1471-4159.2008.05588.x

[22] Sladowski D , Steer SJ , Clothier RH , *et al* An improved MTT assay. J Immunol Methods. 1993; 157: 203–7. 8423364 10.1016/0022-1759(93)90088-o

[23] Paxinos G , Watson C . The Rat Brain in Stereotaxic Coordinates. 4th edn San Diego: Academic Press; 1998.

[24] Choo YS , Zhang Z . Detection of protein ubiquitination. J Vis Exp. 2009; 30: 1293. 10.3791/1293 PMC3149903 19692941

[25] Kalia LV , Lang AE . Parkinson's disease. Lancet. 2015; 386: 896–912. 25904081 10.1016/S0140-6736(14)61393-3

[26] Ferrer I , Blanco R , Carmona M , *et al* Active, phosphorylation‐dependent mitogen‐activated protein kinase (MAPK/ERK), stress‐activated protein kinase/c‐Jun N‐terminal kinase (SAPK/JNK), and p38 kinase expression in Parkinson's disease and Dementia with Lewy bodies. J Neural Transm. 2001; 108: 1383–96. 11810403 10.1007/s007020100015

[27] Heras‐Sandoval D , Perez‐Rojas JM , Hernandez‐Damian J , *et al* The role of PI3K/AKT/mTOR pathway in the modulation of autophagy and the clearance of protein aggregates in neurodegeneration. Cell Signal. 2014; 26: 2694–701. 25173700 10.1016/j.cellsig.2014.08.019

[28] Hetz C . The unfolded protein response: controlling cell fate decisions under ER stress and beyond. Nat Rev Mol Cell Biol. 2012; 13: 89–102. 22251901 10.1038/nrm3270

[29] Yang W , Paschen W . The endoplasmic reticulum and neurological diseases. Exp Neurol. 2009; 219: 376–81. 19616544 10.1016/j.expneurol.2009.07.009

[30] Fouillet A , Levet C , Virgone A , *et al* ER stress inhibits neuronal death by promoting autophagy. Autophagy. 2012; 8: 915–26. 22660271 10.4161/auto.19716 PMC3427257

[31] Michel PP , Hirsch EC , Hunot S . Understanding Dopaminergic Cell Death Pathways in Parkinson Disease. Neuron. 2016; 90: 675–91. 27196972 10.1016/j.neuron.2016.03.038

[32] Colla E , Coune P , Liu Y , *et al* Endoplasmic reticulum stress is important for the manifestations of alpha‐synucleinopathy *in vivo* . J Neurosci. 2012; 32: 3306–20. 22399753 10.1523/JNEUROSCI.5367-11.2012 PMC3461828

[33] Magre J , Delepine M , Khallouf E , *et al* Identification of the gene altered in Berardinelli‐Seip congenital lipodystrophy on chromosome 11q13. Nat Genet. 2001; 28: 365–70. 11479539 10.1038/ng585

[34] Binns D , Lee S , Hilton CL , *et al* Seipin is a discrete homooligomer. Biochemistry. 2010; 49: 10747–55. 21062080 10.1021/bi1013003 PMC3086013

[35] Lundin C , Nordstrom R , Wagner K , *et al* Membrane topology of the human seipin protein. FEBS Lett. 2006; 580: 2281–4. 16574104 10.1016/j.febslet.2006.03.040

[36] Dollet L , Magre J , Cariou B , *et al* Function of seipin: new insights from Bscl2/seipin knockout mouse models. Biochimie. 2014; 96: 166–72. 23831461 10.1016/j.biochi.2013.06.022

[37] Wei S , Soh SL , Qiu W , *et al* Seipin regulates excitatory synaptic transmission in cortical neurons. J Neurochem. 2013; 124: 478–89. 23173741 10.1111/jnc.12099

[38] Wei S , Soh SL , Xia J , *et al* Motor neuropathy‐associated mutation impairs Seipin functions in neurotransmission. J Neurochem. 2014; 129: 328–38. 24345054 10.1111/jnc.12638

[39] Garfield AS , Chan WS , Dennis RJ , *et al* Neuroanatomical characterisation of the expression of the lipodystrophy and motor‐neuropathy gene Bscl2 in adult mouse brain. PLoS One. 2012; 7: e45790. 23049863 10.1371/journal.pone.0045790 PMC3458087

[40] Licker V , Turck N , Kovari E , *et al* Proteomic analysis of human substantia nigra identifies novel candidates involved in Parkinson's disease pathogenesis. Proteomics. 2014; 14: 784–94. 24449343 10.1002/pmic.201300342

[41] Qian Y , Yin J , Hong J , *et al* Neuronal seipin knockout facilitates Abeta‐induced neuroinflammation and neurotoxicity *via* reduction of PPARgamma in hippocampus of mouse. J Neuroinflammation. 2016; 13: 145. 27287266 10.1186/s12974-016-0598-3 PMC4902906

[42] Ruiz‐Riquelme A , Sanchez‐Iglesias S , Rabano A , *et al* Larger aggregates of mutant seipin in Celia's Encephalopathy, a new protein misfolding neurodegenerative disease. Neurobiol Dis. 2015; 83: 44–53. 26282322 10.1016/j.nbd.2015.08.006

[43] Guillen‐Navarro E , Sanchez‐Iglesias S , Domingo‐Jimenez R , *et al* A new seipin‐associated neurodegenerative syndrome. J Med Genet. 2013; 50: 401–9. 23564749 10.1136/jmedgenet-2013-101525

[44] Zhou L , Yin J , Wang C , *et al* Lack of seipin in neurons results in anxiety‐ and depression‐like behaviors *via* down regulation of PPARgamma. Hum Mol Genet. 2014; 23: 4094–102. 24651066 10.1093/hmg/ddu126

[45] Yagi T , Ito D , Nihei Y , *et al* N88S seipin mutant transgenic mice develop features of seipinopathy/BSCL2‐related motor neuron disease *via* endoplasmic reticulum stress. Hum Mol Genet. 2011; 20: 3831–40. 21750110 10.1093/hmg/ddr304

[46] Guo J , Qiu W , Soh SL , *et al* Motor neuron degeneration in a mouse model of seipinopathy. Cell Death Dis. 2013; 4: e535. 23470542 10.1038/cddis.2013.64 PMC3613842

[47] Oh YM , Jang EH , Ko JH , *et al* Inhibition of 6‐hydroxydopamine‐induced endoplasmic reticulum stress by l‐carnosine in SH‐SY5Y cells. Neurosci Lett. 2009; 459: 7–10. 19394406 10.1016/j.neulet.2009.04.047

[48] Blum D , Torch S , Lambeng N , *et al* Molecular pathways involved in the neurotoxicity of 6‐OHDA, dopamine and MPTP: contribution to the apoptotic theory in Parkinson's disease. Prog Neurobiol. 2001; 65: 135–72. 11403877 10.1016/s0301-0082(01)00003-x

[49] Zhao Q , Gao J , Li W , *et al* Neurotrophic and neurorescue effects of Echinacoside in the subacute MPTP mouse model of Parkinson's disease. Brain Res. 2010; 1346: 224–36. 20478277 10.1016/j.brainres.2010.05.018

[50] Zhao Q , Yang X , Cai D , *et al* Echinacoside Protects Against MPP(+)‐Induced Neuronal Apoptosis *via* ROS/ATF3/CHOP Pathway Regulation. Neuroscience Bullet. 2016; 32: 349–62. 10.1007/s12264-016-0047-4 PMC5563786 27432061

[51] Zhang J , Zhang Z , Xiang J , *et al* Neuroprotective Effects of Echinacoside on Regulating the Stress‐Active p38MAPK and NF‐kappaB p52 Signals in the Mice Model of Parkinson's Disease. Neurochem Res. 2017; 42: 975–85. 27981472 10.1007/s11064-016-2130-7

[52] Jia C , Shi H , Wu X , *et al* Determination of echinacoside in rat serum by reversed‐phase high‐performance liquid chromatography with ultraviolet detection and its application to pharmacokinetics and bioavailability. J Chromatogr B Analyt Technol Biomed Life Sci. 2006; 844: 308–13. 10.1016/j.jchromb.2006.07.040 16931184

[53] Zhou J , Zeng P , Sun JB , *et al* Application of two‐phase hollow fiber liquid phase microextraction coupled with high‐performance liquid chromatography for the study of the echinacoside pharmacokinetics in Parkinson's disease rat plasma. J Pharm Biomed Anal. 2013; 81–82: 27–33. 10.1016/j.jpba.2013.03.020 23591053

[54] Shen JY , Yang XL , Yang ZL , *et al* Enhancement of absorption and bioavailability of echinacoside by verapamil or clove oil. Drug Des Devel Ther. 2015; 9: 4685–93. 10.2147/DDDT.S87581 PMC4544722 26316707

[55] Tanino T , Nagai N , Funakami Y . Phloridzin‐sensitive transport of echinacoside and acteoside and altered intestinal absorption route after application of Cistanche tubulosa extract. J Phar Pharmacol. 2015; 67: 1457–65. 10.1111/jphp.12450 26179928

