# Supplementary material for: Echinacoside's nigrostriatal dopaminergic protection against 6‐OHDA‐Induced endoplasmic reticulum stress through reducing the accumulation of Seipin
Source: J Cell Mol Med. 2017 Aug 2;21(12):3761–75. doi: 10.1111/jcmm.13285 (PMC5706584; doi:10.1111/jcmm.13285)
Supplement: Supplementary file 1 — Figure S1. Operative procedure and injection on the rat model. (a) vertical view (b) lateral view. Figure S2. The whole procedure of animal experiments. [file JCMM-21-3761-s001.docx]

# Supplementary


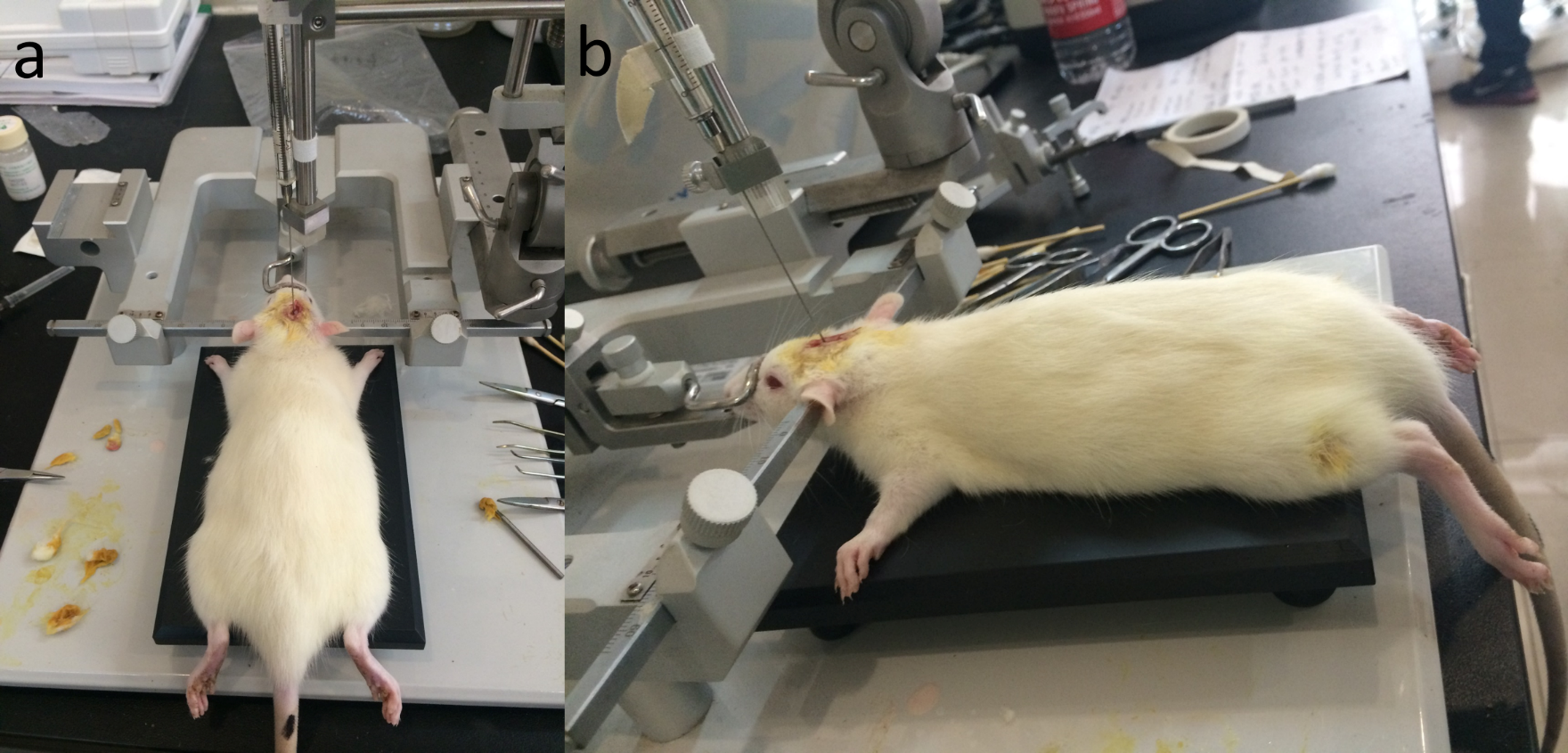


**Figure S1.** **Operative procedure and injection on the rat model.** (**a**) vertical view (**b**) lateral view


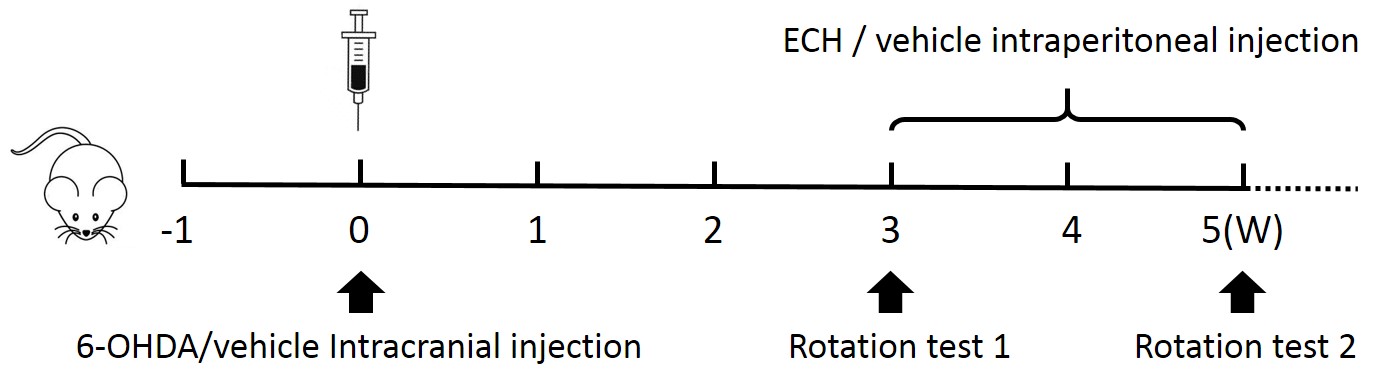


**Figure S2. The whole procedure of animal experiments.**

Supplementary video: The typical rotation behavior of rats.MOV 68.6 MB

Time: 00:00:33
